# Comprehensive management of immune-related hemolytic anemia in a patient with acute cholangitis: A case report

**DOI:** 10.1097/MD.0000000000045892

**Published:** 2025-11-21

**Authors:** Jun Chen, Xian-Feng Wang, Jin Yu, Sheng Xu, Feng Zheng, Zhong-Kai Ni

**Affiliations:** a Department of Critical Care Medicine, Hangzhou Lin’an Traditional Chinese Medicine Hospital, Hangzhou, Zhejiang Province, China; b Department of General Surgery, Hangzhou Lin’an Traditional Chinese Medicine Hospital, Hangzhou, Zhejiang Province, China; c Department of Clinical Laboratory, Hangzhou Lin’an Traditional Chinese Medicine Hospital, Hangzhou, Zhejiang Province, China; d Department of General Surgery, Hangzhou Hospital of Traditional Chinese Medicine, Hangzhou, Zhejiang Province, China.

**Keywords:** acute cholangitis, choledocholithiasis, endoscopic retrograde cholangiopancreatography, immune-associated hemolytic anemia, perioperative treatment

## Abstract

**AbstractRationale:**

Immune-associated hemolytic anemia is an uncommon and potentially life-threatening complication in patients with choledocholithiasis and acute cholangitis. Its occurrence during the perioperative period remains rarely reported.

**Patient concerns:**

A 69-year-old woman presented with right upper abdominal pain and was diagnosed with choledocholithiasis and acute cholangitis. During hospitalization, she developed sudden confusion and shock, with her hemoglobin rapidly declining to 26 g/L.

**Diagnoses:**

The patient was diagnosed with acute cholangitis and immune-related hemolytic anemia based on clinical manifestations and a positive direct antihuman globulin (Coombs) test.

**Interventions:**

The patient received high-dose glucocorticoid therapy combined with red blood cell transfusion, followed by endoscopic retrograde cholangiopancreatography with endoscopic sphincterotomy and nasobiliary drainage to relieve biliary obstruction, and subsequent laparoscopic cholecystectomy.

**Outcomes:**

After timely diagnosis and comprehensive management, the patient’s hemoglobin stabilized, hemolysis resolved, and she recovered without postoperative complications.

**Lessons:**

This case provides 3 important lessons for clinical practice: The need for prompt recognition and management of unexpected perioperative complications and flexible adjustment of clinical thinking. A systematic approach for handling sudden and life-threatening hemoglobin decline. Appropriate timing and optimal surgical strategy selection for delayed definitive surgery after stabilization.

## 1. Introduction

The diagnosis and treatment of immune-associated hemolytic anemia have long been considered difficult and complex. Due to the hidden nature of its onset, rapid and accurate clinical judgment is crucial for initiating treatment. However, making a differential diagnosis through the “unique” clues of clinical symptoms and laboratory tests at an early stage is challenging. Here, we report a patient with choledocholithiasis complicated by acute cholangitis who was awaiting treatment within a limited surgical window. The patient developed very rare immune-associated hemolytic anemia during preoperative preparation and treatment. During the onset of the disease, the patient experienced severe shock and circulatory failure. After a rapid and accurate diagnosis, we managed the hemolytic anemia and completed 2 minimally invasive interventional operations at the most appropriate time, achieving excellent primary disease treatment outcomes. Eventually, the elderly patient with underlying diseases recovered. Among the current literature reports, all the patients with fatal hemolytic anemia caused by anti-infective drugs were not during the perioperative period, and no surgical treatment was required within a specific time frame. This also highlights the unique value of this individual case.

## 2. Case presentation

### 2.1. Chief complaints

A 69-year-old woman born in Lin ‘an presented with right upper abdominal pain that persisted for 8 hours.

### 2.2. History of present illness

The patient reported pain in the right upper abdomen at approximately 14:00 on the same day without obvious causes, which was slightly relieved after rest, without fever, chills, nausea, vomiting, dizziness, headache, palpitations, chest tightness, chest pain, low back pain, hematuria, diarrhea, or stopped anal exhaust and defecation. The pain gradually got worse during dinner.

### 2.3. History of past illness

The patient had preexisting hypertension and diabetes, both of which were controlled by medication.

### 2.4. Personal and family history

The patient denied the living history or contact history of infected water in the epidemic area, as well as any history of drug allergy, hereditary disease, congenital disease, or other infectious diseases in the family. The patient was a nonsmoker and did not use alcohol. Both parents died of unknown causes.

### 2.5. Physical examination

Upon admission, the patient exhibited right upper abdomen tenderness, no rebound pain, liver and spleen that could not be reached under the ribs, no abdominal mass, Murphy sign of gallbladder weak positive, and double kidney percussion pain negative.

### 2.6. Laboratory examinations

On day 1, her blood test results were as follows: total bilirubin 124.6 μmol/L↑, direct bilirubin 86.9 μmol/L↑, alanine aminotransferase 569 U/L↑, aspartate aminotransferase 1066 U/L↑.

### 2.7. Imaging examinations

**Figure 1.**
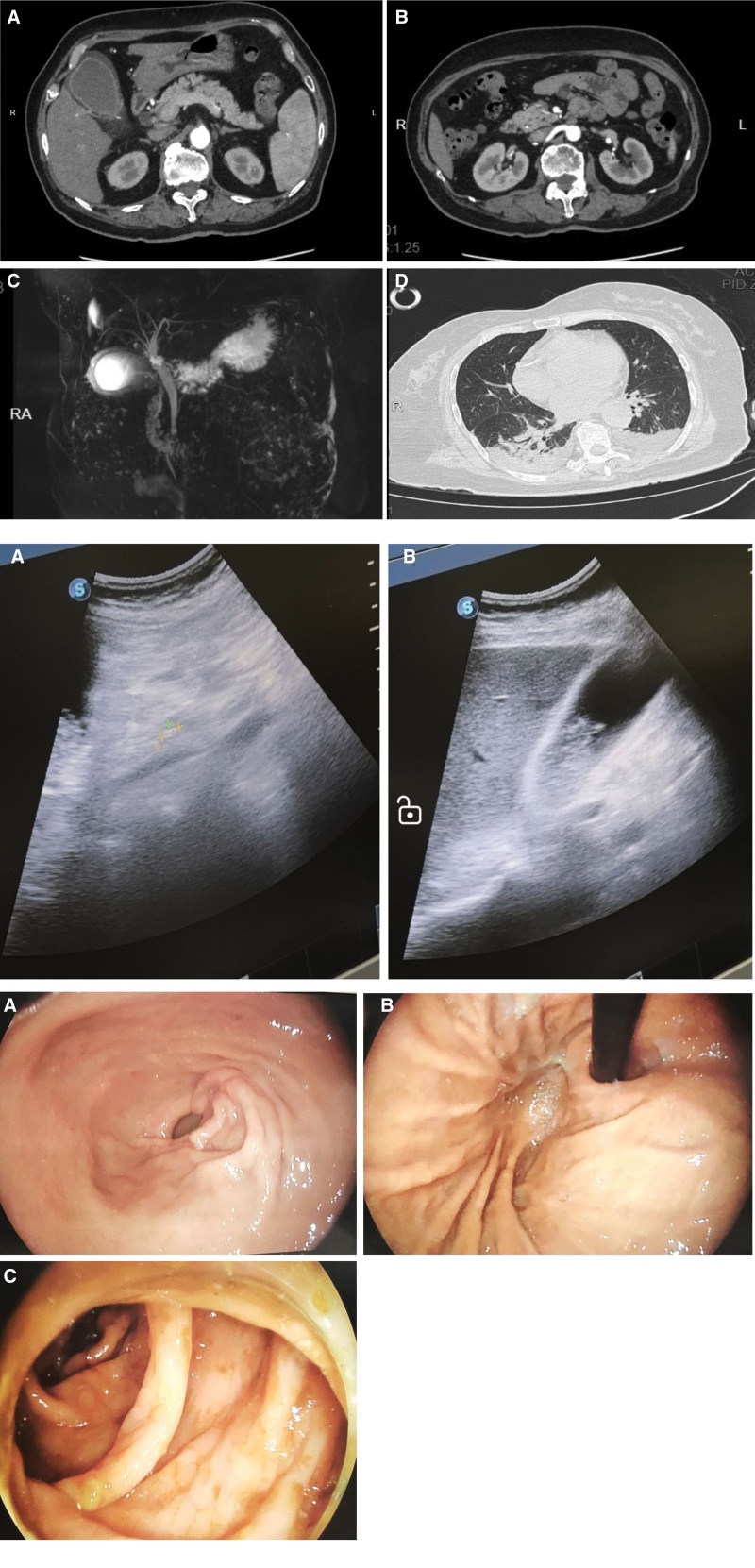
(A. A) Enhanced abdominal CT on March 8, 2024, showed significant cystic edema. (B) Enhanced abdominal CT on March 8, 2024: the common bile duct was dilated and a stone shadow was visible at the lower end. (C) Abdominal magnetic resonance cholangiopancreatography on March 10, 2024: common bile duct dilatation with choledocholithiasis at the lower end of the common bile duct. (D) Lung CT on March 11, 2024: bilateral posterior lower lobe inflammation with pleural effusion. (B. A) Bedside ultrasound on March 11, 2024, revealed a large number of silt-like stones in the gallbladder. (B) Bedside ultrasound on March 11, 2024, which indicated significant bile duct dilatation. (C. A) On March 11, 2024, bedside gastroscope showed no significant bleeding in the antrum. (B) On March 11, 2024, bedside gastroscope showed no significant bleeding in the fundus of the stomach. (C) After bedside colonoscopy on March 11, 2024, no significant bleeding was observed in the colon.

The patient was hospitalized on March 5, 2024, due to right upper abdominal pain, and based on upper abdominal computed tomography (CT), gallstones with cholecystitis were considered. Choledochal dilatation, possible lower choledochal calculus, and acute cholangitis were diagnosed. On March 8, 2024, total abdominal enhanced CT did not find tumor space in the lower segment of the common bile duct, the pancreas, and the surrounding ampulla, and magnetic resonance cholangiopancreatography showed full dilation of the common bile duct with lower segment stones on March 10, 2024 (Fig. 1A).

From March 10, 2024, to March 11, 2024, in case of a persistent decline in hemoglobin, we began to look for active bleeding. Bedside ultrasound indicated no effusion in the thoracic, pericardial, abdominal, and pelvic cavities, no bleeding was observed in gastric tube drainage, and ultrasound showed cholecystolithiasis with bile duct dilation (Fig. 1B). The fecal occult blood test was negative, and bedside gastroenteroscopy showed no significant active bleeding (Fig. 1C). The dynamic assessment of the cause of hemoglobin decline continued. The patient underwent head and chest CT scans (March 11, 2024) immediately after hemodynamic stabilization; no intracranial hemorrhage was observed, and inflammation with pleural effusion was observed in both lungs.

## 3. Initial treatment and medication

The patient was treated with 40 mg of phloroglucinol in the emergency department for acute cholangitis, which slightly relieved the right upper abdominal pain. After admission, the patient was administered an intravenous drip of 2.0 g ceftriaxone sodium injection, 150 mg magnesium isoglycyrrhizinate injection for liver protection, and 80 mg phloroglucinol injection for spasmolytic treatment. Due to a long history of coronary heart disease, the patient had been taking one tablet of aspirin daily, which was suspended upon admission and replaced with a subcutaneous injection of 2500 IU Dalteparin Sodium injection once a day.

## 4. Rescue, diagnosis, and treatment

On the fifth day of admission, the jaundice had slightly worsened compared to that at admission. Blood tests showed: total bilirubin 66.2 μmol/L↑, direct bilirubin 49.2 μmol/L↑, alanine aminotransferase 144 U/L↑, the ratio of glutamyl-grass to alanine ↑ 0.22↓, gamma-glutamyl transferase 387 U/L↑, erythrocyte 2.90 × 10^12^/L↓, hemoglobin 83 g/L↓, hematocrit 24.00%↓, and fibrinogen 4.58 g/L↑.

On the sixth day of hospitalization, blood examination revealed the following: glucose 14.6 mmol/L, white blodd cells (WBCs) 11.1 × 10^9^/L↑, red blood cells (RBCs) 0.37 × 10^12^/L↓, hemoglobin 26 g/L↓, and D-dimer (quantitative) 2480 ng/mL↑. Arterial blood gas results showed a pH of 7.458↑, partial pressure of oxygen 136.8 mm Hg↑, partial pressure of carbon dioxide 24.6 mm Hg↓, and whole blood lactic acid 6.5 mmol/L↑. At approximately 9:10 am, the patient went out for a magnetic resonance cholangiopancreatography examination and suddenly became unconscious after returning to the ward. The patient did not respond to calls, and these symptoms lasted for approximately 2 minutes before regaining consciousness. Subsequently, the patient was extremely weak and vomited once, expelling watery gastric contents. The patient’s complexion was pale and jaundiced, the pupils were equal in size and round, reactive to light, the muscle strength of the limbs was IV+, and symmetrical on both sides, with no pathological signs. Oxygen was administered via Venturi mask at 50% FiO_2_, maintaining SpO_2_ between 90% and 95%. Blood pressure dropped as low as 90/55 mm Hg, and glucose was measured at 14.6 mmol/L. The patient was immediately given crystal solution (Ringer’s fluid) for fluid supplementation and antishock therapy.

**Figure 2.**
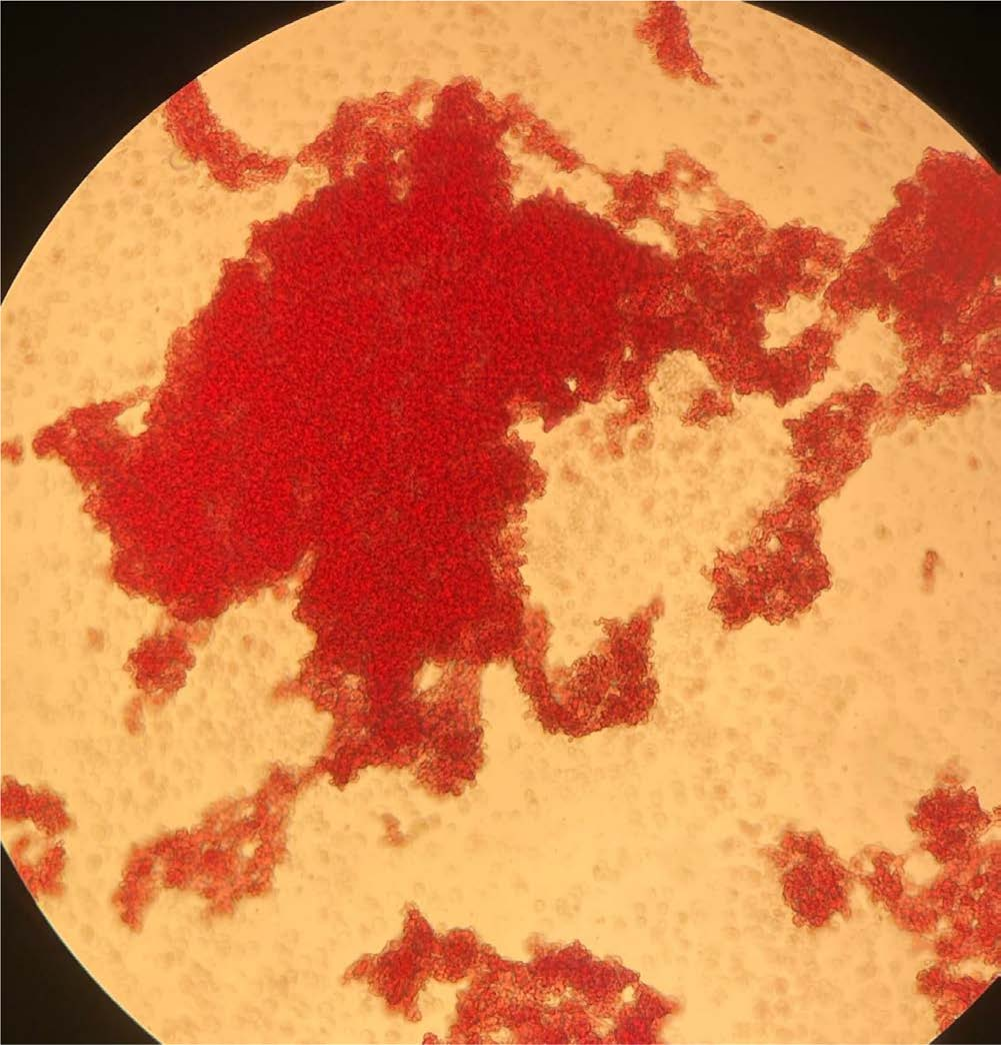
A blood smear taken at 10:00 on March 10, 2024, showed significant RBC aggregation and surrounding ruptured RBCs. RBC = red blood cell.

**Figure 3.**
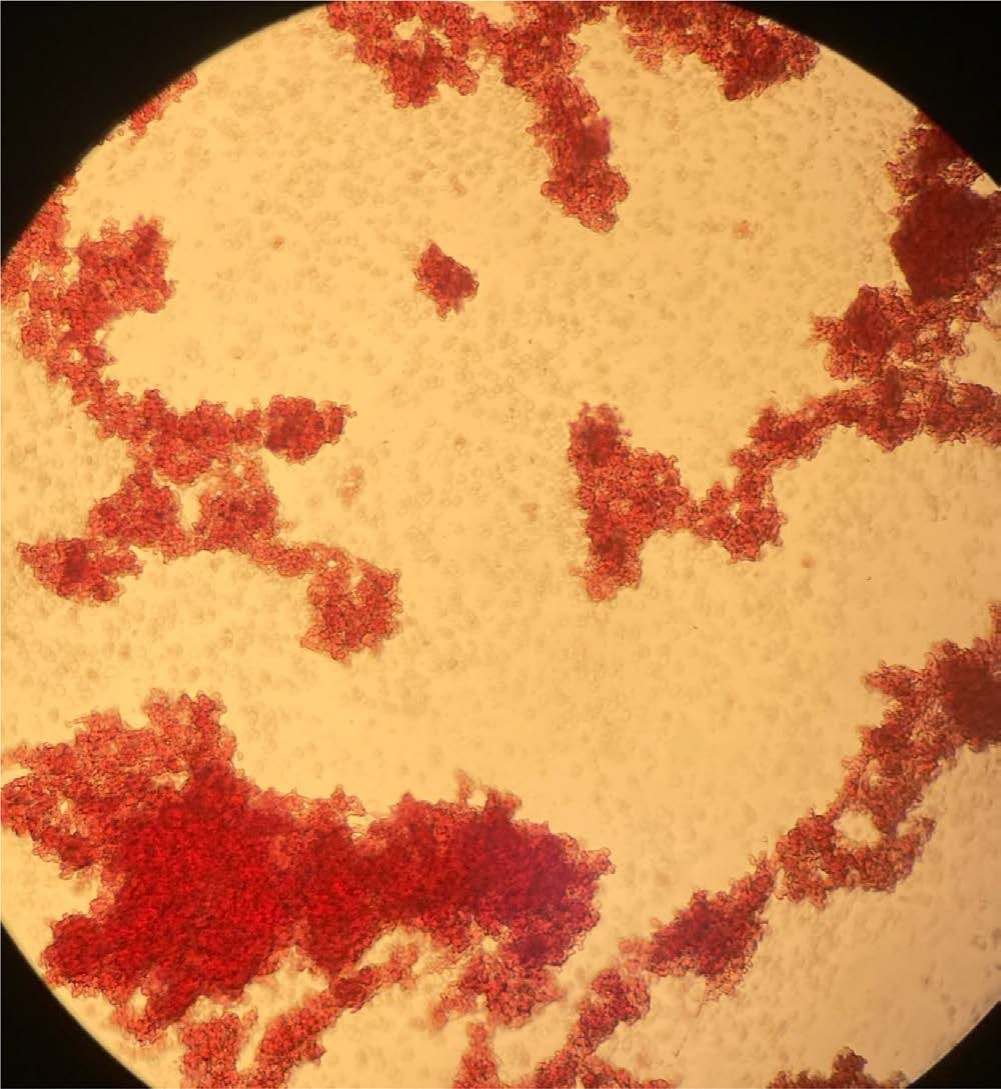
A blood smear taken on March 10, 2024, at 11:00, showed that RBC were aggregated into clumps. RBC = red blood cell

The patient was emergently transferred from the ward to the intensive care unit for continued treatment. The patient’s blood pressure continued to drop sharply, reaching a minimum of 60/30 mm Hg. Rapid fluid resuscitation was administered, and the mean arterial pressure was maintained with norepinephrine. The highest dose of norepinephrine reached 0.09 μg/kg/min. The patient was urgently transferred to the intensive care unit, where arterial blood gas showed whole blood lactic acid at 11.1 mmol/L↑. A blood test reported hemoglobin levels at 24 g/L↓ and noted a RBC agglutination phenomenon.The blood bank was immediately asked to prepare 6 units of RBC suspension and 600 mL of type O, Rh-positive plasma. However, the blood transfusion department found severe RBC agglutination, making cross-matching impossible. Therefore, the blood transfusion department used a blood sample from March 9, 2024, for cross-matching, and the blood was successfully transfused. At 10:00 am and 11:00 am on March 10, 2024, the laboratory once again confirmed whether there were errors in the recognition of RBCs and hemoglobin by the machine. It was found that a large number of RBCs formed clumps, and there were ruptured RBCs around them (Fig. 2 and Fig. 3).

### 4.1. Comprehensive treatment

**Figure 4.**
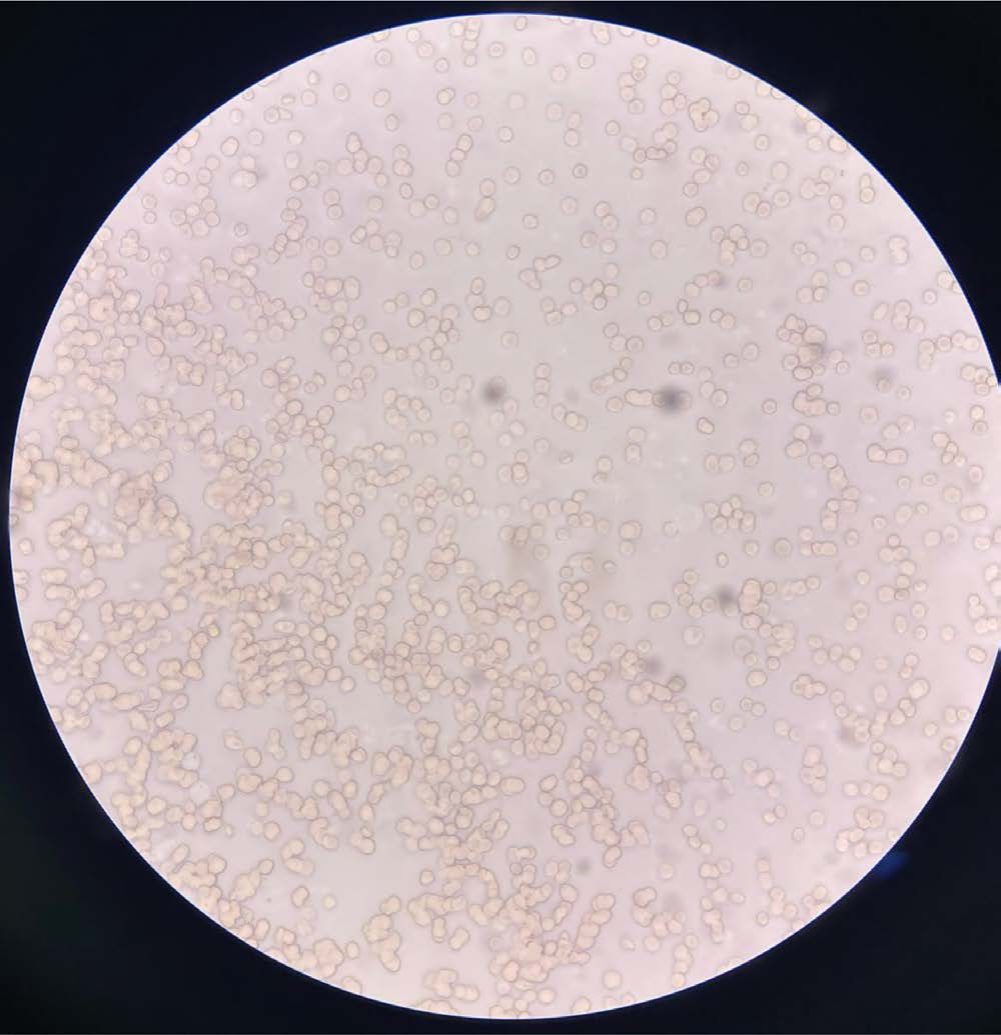
In the blood smear taken at 6:17 pm on March 11, 2024, after hormone and blood transfusion treatment, there was no obvious RBC mass aggregation under the microscope. RBC = red blood cell.

On March 11, 2024, laboratory results showed the following: RBC count 3.32 × 10^12^/L↓, hemoglobin 100 g/L↓, lymphocyte percentage 10.5%↓, high-sensitivity C-reactive protein 33.96 mg/L↑, and reticulocyte count % 4.0%↑; no RBC agglutination was found (Fig. 4). The pH value was 7.398, the partial pressure of oxygen was 91.5 mm Hg, the partial pressure of carbon dioxide was 30.7 mm Hg↓, the lactic acid was 1.4 mmol/L, bicarbonate was 18.5 mmol/L↓, and the base excess was − 5.00 mmol/L. Serum amyloid protein was 120.6 mg/L↑, and D-dimer (quantitative) was 13,790 ng/mL. Mechanical assisted ventilation with tracheal intubation was initiated. Gastroscopy and colonoscopy were also performed, revealing no significant active gastrointestinal bleeding.Laboratory findings indicated the following (March 13, 2024): erythrocyte incubation osmotic brittleness test (colorimetric method), 93%; glucose-6-phosphate dehydrogenase defect screening (colorimetric method), 1.91 (normal range: 1.00–2.30); direct antihuman globulin test using the microcolumn gel method, positive (+); indirect antihuman globulin test for erythrocyte agglutination, negative (-); plasma free hemoglobin determination, colorimetric method > 800.00 mg/L ↑ (normal range: <40.00) and immunoturbidimetric method < 25.00 mg/dL↓ (normal range: 30.00–200.00); hemoglobin A via capillary electrophoresis, 97.7% ↑ (94.5–97.5); hemoglobin F via capillary electrophoresis, 0.0% (0.0–2.0); hemoglobin A2 (HbA2) via capillary electrophoresis, 2.3% ↓ (2.5–3.5); other hemoglobin variants: none detected; and hemoglobin H inclusion body detection, none.

## 5. Final diagnosis

The final diagnosis included the following: immune hemolytic anemia, choledocholithiasis with acute cholangitis, obstructive jaundice, hemolytic jaundice, severe anemia, shock, and gallstones with acute cholecystitis.

## 6. Outcome and follow-up

**Figure 5.**
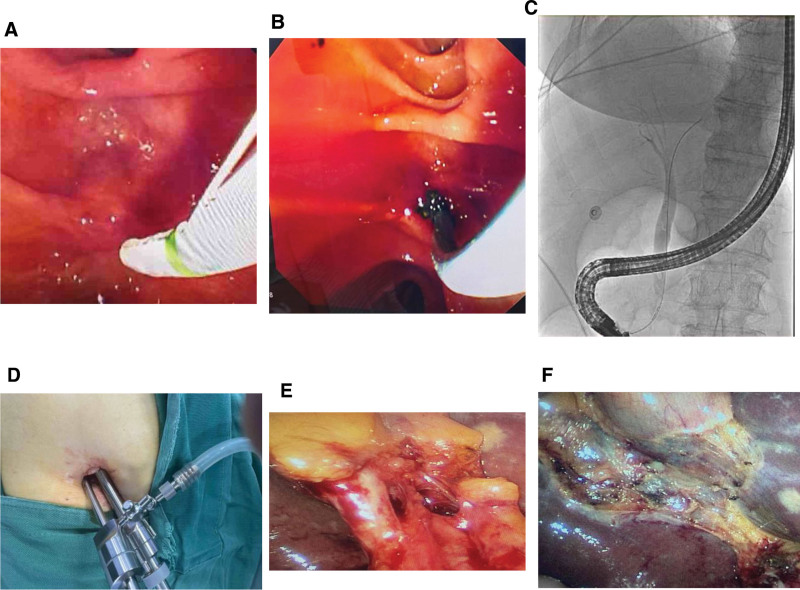
(A) Incision of the duodenal papilla under ERCP side view mirror. (B) The stones at the lower end of the common bile duct were removed with a lithotomy basket under ERCP. (C) ERCP retrograde cholangiography. (D) Double channel insertion of the puncture device was performed around the umbilicus. (E) Fine dissection of the gallbladder triangle under the endoscopic field of view showed no uncontrolled bleeding. (F) Intraoperative gallbladder bed condition, no obvious bleeding. ERCP = endoscopic retrograde cholangiopancreatography.

**Figure 6.**
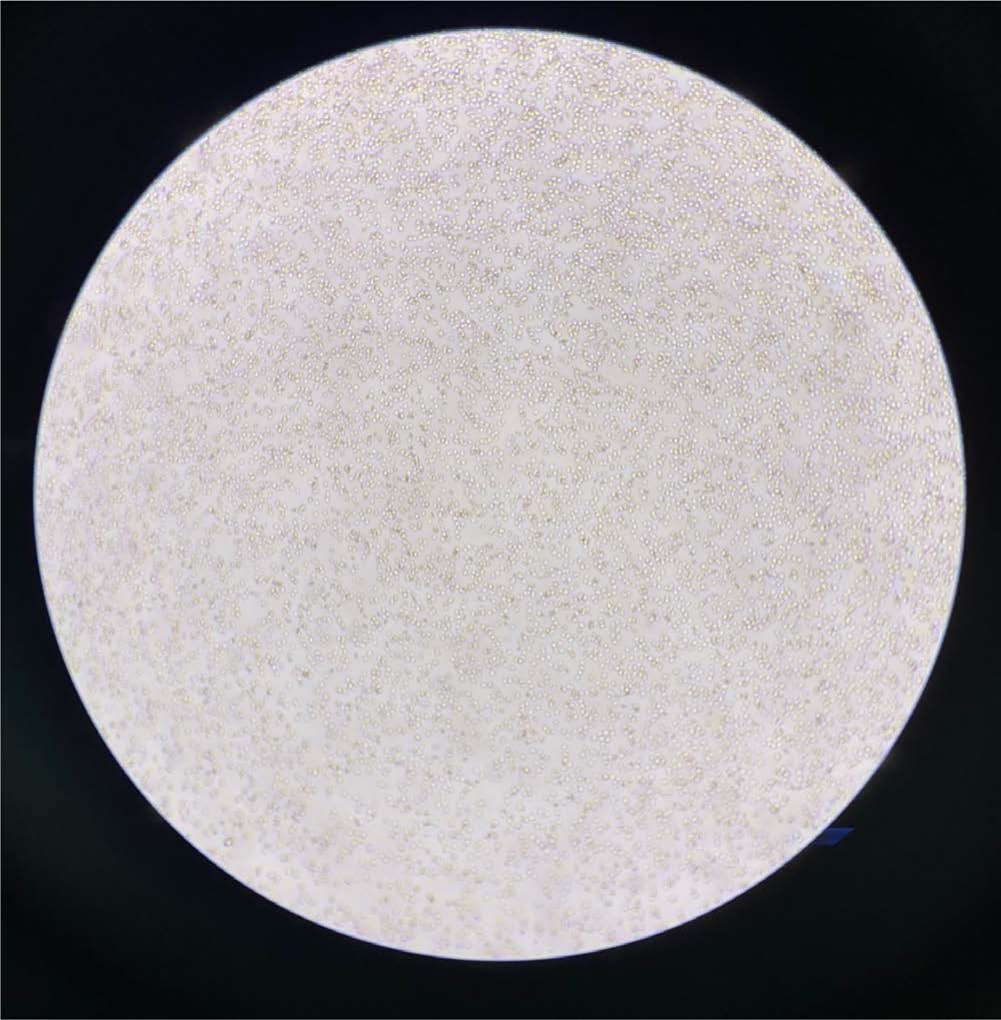
Blood routine review before laparoscopic cholecystectomy; blood smear at 8:26 on March 19, 2024, showed no obvious RBC mass aggregation under the microscope. RBC = red blood cell.

**Figure 7.**
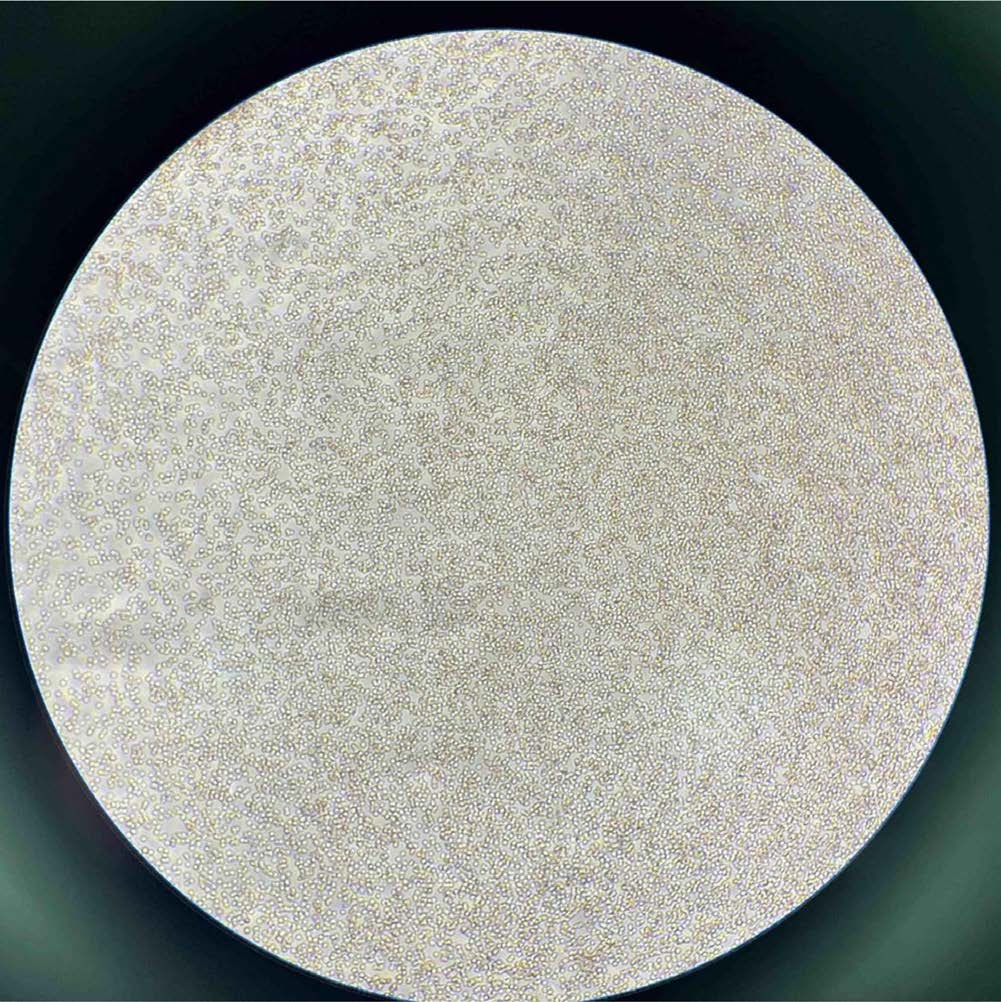
Blood routine review after laparoscopic cholecystectomy; blood smear at 11:00 am on March 21, 2024, showed no obvious RBC mass aggregation under the microscope. RBC = red blood cell.

On the same day, the patient underwent endoscopic retrograde cholangiopancreatography + endoscopic sphincterotomy + endoscopic nasobiliary drainage in the cath lab under pharyngeal and intravenous anesthesia due to persistent bile duct inflammation. A small amount of blood seepage was observed at the papilla but was controllable and the whole process of stone removal was without complications (Fig. 5). Postoperative blood tests revealed: WBCs 11.8^9^/L↑, RBCs 3.1 × 10^12^/L↓, hemoglobin 95 g/L↓, total bilirubin 34.8 μmol/L↑, and direct bilirubin 23.4 μmol/L↑ (Fig. 6). On March 20, 2024, a 2-hole laparoscopic cholecystectomy was performed on the patient. The patient experienced cholangitis, shock, and a low hemoglobin state, and coagulation and hemostasis were gradually recovering. The hemostasis and closure of the posterior branch of the gallbladder vessel should be as thorough as possible, and the gallbladder should be removed close to the gallbladder wall at the level to avoid damage to the capillaries on the liver surface resulting in postoperative bleeding (Fig. 5). Postoperative blood tests were as follows: WBCs 5.11 × 10^9^/L, RBCs 3.311 × 10^12^/L↓, hemoglobin 101 g/L↓, total bilirubin 30.8 μmol/L↑, and direct bilirubin 16.3 μmol/L↑ (Fig. 7, Table 1, Table 2).

**Table 1:** Daily test results during the treatment cycle.

	Day 1	Day 5	Day 6	Day 7	Day 8	Day 15	Day 16	Day 19	Day 47	Reference range
WBC (10^9^/L)	6.7	4.6	11.1	14.4	11.8	4.0	5.1	4.1	3.5	3.5–9.5
Norepinephrine (%)	60	63.4	45.6	81.6	81.8	57.5	78.1	65.9	55.9	40–75
RBC (10^12^/L)	4.34	2.90	0.37	3.32	3.10	3.32	3.31	3.20	3.16	3.8–5.1
Hemoglobin (g/L)	125	83	26	100	95	100	101	97	95	115–150
PCT (10^9^/L)	189	155	132	200	164	161	168	148	171	125–350
RET (%)	-	-	-	4.0	-	-	-	-	-	0.5–1.5
ALT (µ/L)	153	144	100	131	78	33	45	29	15	7–40
AST (µ/L)	338	31	130	172	46	26	43	23	20	13–35
Total bilirubin (µmmol/L)	124.6	66.2	88.2	58.7	34.8	28.3	30.8	23.1	27.7	<21
Direct Bilirubin (ummol/L)	86.9	49.2	-	29.1	23.4	18.3	16.3	13.2	13.6	<8
Creatinine (ummol/L)	54	48	46	51	46	65	58	58	45	41–81
ALP (µ/L)	237	216	137	175	159	128	98	100	86	50–135
LDH µ/L)	996	248	1099	1070	731	260	223	212	176	10–250
Albumin (g/L)	37.5	31.8	36.9	35.8	33.7	35.9	34.9	34.3	45.6	40–55
Blood lactic acid (µmmol/L)	-	-	11.1	1.4	1.0	-	-	-	-	0–1.5
PaO_2_ (mm Hg)	-	-	274.3 (FiO_2_100%)	91.5 (FiO_2_40%)	104.2 (FiO_2_33%)	-	-	-	-	80–100
D-Dimer (ng/L)	440	-	2480	13,790	2760	2130	2350	2060	260	<500
Fibrinogen (g/L)	-	4.58	3.89	3.75	2.93	2.61	2.97	3.28	3.02	2.0–4.0
PT (s)	12.7	12.6	13.7	14.2	12.8	13.5	13.3	13.4	13.1	11–14.5
APTT (s)	34.7	39.5	39.1	36.1	32.7	38.1	37.7	37.6	41.8	26–45

ALT = alanine aminotransferase, AST = aspartate aminotransferase, LDH = lactate dehydrogenase, PCT = platelet count, RBC = red blood cell, RET = reticulocyte count, WBC = white blood cell.

**Table 2:** Timeline of the case.

Date	A brief description of the general clinical situation	Presentation of objective laboratory indicators	Summary of current diagnosis and processing
March 5, 2024 (day 1)	The patient was admitted with choledocholithiasis and cholangitis. Right upper abdominal pain, not intense, tolerable, slightly relieved after rest.	Hemoglobin 125 g/L, D-dimer (quantitative) 440 ng/mL, ALT 153 U/L↑, AST 338 U/L↑, upper abdominal CT: gallstones with cholecystitis were considered; Choledochal dilatation, possible lower choledochal calculus.	Considering acute cholangitis, the patients should be treated with spasmolytic, anti-infection and liver protection.
March 9, 2024 (day 5)	The patient’s mental state was significantly improved, the right upper abdominal pain was relieved, and the appetite was gradually improved, but the skin sclera was significantly yellow and deepened.	Total bilirubin 66.2 μmol/L↑, direct bilirubin 49.2 μmol/L↑, alanine transaminase 144 U/L↑, hemoglobin 83 g/L↓, hematocrit 24.00%↓, hypersensitive C-reactive protein 51.47 mg/L↑, fibrinogen 4.58 g/L↑	The patient’s hemoglobin decreased, and the cause of anemia was analyzed. At present, the cholangitis has been controlled. After improving the preoperative evaluation, the bile duct obstruction should be relieved as soon as possible.
March 10, 2024 (day6)	The patient presented with unconsciousness, no response to calls, pale face, yellow sclera of the skin, large and round bilateral pupils, pupils responsive to light, muscle strength IV + in the limbs, and blood pressure decreased to a minimum of 90/55mmHg	RBCs 0.37 × 10^12^/L↓, hemoglobin 26 g/L↓ to 24 g/L↓, D-dimer (quantitative) 2480 ng/ml↑, whole blood lactic acid 6.5 mmol/L↑ to 11.1 mmol/L↑, bedside ultrasound did not show chest and abdominal bleeding	Currently diagnosed as severe anemia, hemorrhagic shock, massive gastrointestinal bleeding? Norepinephrine intravenous micropump maintenance, up to 0.09ug.kg/min. Transfusion treatment: O type, RH (+) RBC suspension 6U, plasma 600ml. Methylprednisolone 40mg was injected intravenously before transfusion.
March 11, 2024 (day 7)	Clear consciousness, nasal catheter oxygen inhalation, skin sclera yellowing better than before, anemia appearance improved.	Hemoglobin 100 g/L↓, whole blood lactic acid 1.4 mmol/L, D-dimer (quantitative) 13,790 ng/mL	Bedside gastroenteroscopy was performed in the morning after tracheal intubation mechanically assisted ventilation. ERCP + EST + ENBD was performed in the afternoon under pharyngeal anesthesia and intravenous anesthesia.
March 20, 2024 (day 16)	There were no obvious abdominal symptoms and signs, and the vital signs were stable	WBCs 5.11 × 10^9^/L, RBCs 3.311 × 10^12^/L↓, hemoglobin 101 g/L↓, total bilirubin 30.8 μmol/L↑, direct bilirubin 16.3 μmol/L↑	Laparoscopic cholecystectomy was performed.

ALT = alanine aminotransferase, AST = aspartate aminotransferase, RBC = red blood cell, WBC = white blood cell.

## 7. Patient perspective

After regaining consciousness, the patient was able to cooperate with drug and surgical treatments. The postoperative pain after endoscopic retrograde cholangiopancreatography and laparoscopic surgery was not severe, allowing the patient to moderately carry out daily activities. Moreover, there were no obvious symptoms such as chest tightness, palpitations, or shortness of breath after exercise due to anemia.

## 8. Discussion

Hemolytic anemia is usually evaluated early through symptoms such as weakness, abdominal pain, and jaundice. Laboratory indicators include decreased hemoglobin, decreased haptoglobin, elevated lactate dehydrogenase, hyperbilirubinemia, and positive direct Coombs tests.^[1]^ However, cholangitis, a condition the patient already had, can also manifest similar signs and symptoms, as well as comparable laboratory results. Bianco *et al* found that there was significant interference in the diagnosis of autoimmune hemolytic anemia in patients with liver diseases,^[2]^ and Gonzalez-Moreno *et al* found that there was a correlation between primary cholangitis and warm autoimmune hemolytic anemia.^[3]^ This uniqueness prompted us to share this case. The causes of hemolytic anemia are varied, and drug-induced causes are relatively rare. Ceftriaxone has been reported to cause drug-induced immune-related hemolytic anemia. Moreover, most of the relevant literature focuses on the hemolytic reactions caused by drugs after the treatment of internal infections. In this case, the hemolytic reaction occurred during the perioperative period and required a timely surgical intervention in the future. This also highlights the unique value of this presentation. Nonetheless, we maintain our belief in the overall safety and efficacy of ceftriaxone as a crucial antibiotic for biliary tract infections. The immune response time of this patient was indeed consistent with drug-induced immune-related hemolysis.^[4,5]^ However, due to the patient’s own obstructive suppurative cholangitis, there is the possibility of initiating inflammatory storms, especially considering the patient’s chronic underlying disease. The administration of both oral and intravenous drugs during treatment further complicates the scenario, making it challenging to completely rule out the possibility of other drugs or factors inducing immune hemolysis. Moreover, some studies suggest that infections themselves can also induce immune-related hemolysis.^[6]^

Indeed, drug-related autoimmune-related hemolytic anemia is rare but can lead to serious complications. It is characterized by drug-mediated immune response, where some drugs or their metabolites act as haptens, binding to proteins on the erythrocyte membrane and forming complete antigens. This process can lead to lipid peroxidation of the erythrocyte membrane, rendering it unstable.^[7]^ Alternatively, drugs can directly combine with antibodies in the blood to form immune complexes that activate the complement system. The activated products of complement can insert into the erythrocyte membrane and destroy its stability.^[8]^ Furthermore, a drug may induce the activation of the body’s T cells, producing an immune response against RBCs.^[9]^ Consequently, immune-mediated erythrocytes bound to the antibodies are engulfed by macrophages in the spleen, resulting in hemolysis. A crucial step in early diagnosis is to distinguish between cases caused by drug-dependent antibodies and those caused by drug-independent antibodies. This differentiation is essential for guiding appropriate treatment strategies.^[10]^

The significant and rapid decrease in RBCs and hemoglobin in this case can be attributed to the agglutination and clumping of RBCs, which cannot be automatically detected using routine blood analyzers and arterial blood gas analyzers. These agglutinated RBCs are temporarily rendered inactive, but they may not have had enough time to be destroyed and broken down. The resulting anoxic state in the microcirculation caused by the agglutination of a large number of RBCs is also a contributing factor to the patient’s rapid shock state. However, after timely steroid administration and circulatory support, peripheral blood smears revealed no significant presence of agglutinated RBCs.^[11]^ Instead, fragments formed after the inactivation or destruction of RBCs due to agglutination were observed within a short period. Therefore, it was crucial to quickly “interrupt” the immune storm and effectively restore the oxygen carrier.^[12]^ Without timely detection or intervention, these agglutinated RBCs may continue to be destroyed rapidly over a short period. Studies have found that patients without timely treatment may develop serious intravascular hemolysis complications, including acute renal failure and disseminated intravascular coagulation.^[13]^

In cases where the cause cannot be promptly identified or when there is no rapid promotion of endogenous RBCs, effective infusion of exogenous RBCs is the most direct approach. In this case, when obtaining washed RBCs promptly was not feasible, we opted for packed RBCs with WBCs removed. Re-administration of glucocorticoids before transfusion minimizes the occurrence of transfusion reactions and reduces the immune response to foreign blood, especially when it is in a hyperimmune state. In this case, the patient had provided a blood sample the day before the onset of hemolysis, which ultimately facilitated the completion of cross-matching for the patient. We understand the challenges associated with accurately matching blood for such patients, and obtaining a sample prior to the onset of complications proved to be fortuitous in ensuring a successful transfusion.

Indeed, a unique feature of this patient’s case is that even after the occurrence of hemolysis or shock, the primary disease remains inadequately treated, requiring surgical intervention by a surgeon, often under limited or emergency conditions. The profound shock caused by hemolytic anemia significantly impacts the organ function of patients, especially the liver and kidney functions. The choice of surgical timing and dynamic evaluation are also very important.^[14]^ Ill-timed surgery can trigger a more severe immune storm again, leading to an irreversible and fatal outcome. In this case, we decided to relieve the biliary obstruction immediately after resuscitation. Indeed, ceftriaxone has a good therapeutic effect on biliary tract infections caused by gram-negative bacilli. Timely detection of the coagulation function and erythrocyte condition during treatment is helpful for the timely detection of such dangerous diseases as immune hemolysis.

Although hemolytic anemia itself may not significantly impair coagulation function, the massive release of hemoglobin from erythrocyte destruction can affect the coagulation mechanism. Additionally, tissue hypoxia resulting from the anemic state can disrupt coagulation balance.^[15]^ Furthermore, the immune storm associated with hemolytic anemia may affect the hemostatic function of platelets, increasing the risk of bleeding. Therefore, for both of our subsequent follow-up surgeries, we chose the precise and minimally invasive approach to complete them.

## 9. Conclusion

In the treatment of immune-related hemolytic anemia, the critical focus lies in identifying possible immune triggers and quickly interrupting the immune storm. We believe that early, adequate administration of glucocorticoids is effective, but it is crucial to evaluate the patient’s primary disease condition beforehand, especially in patients with contraindications to steroid use. The choice of better immunosuppressants is also a matter of more in-depth future research. To address the microcirculation hypoxia caused by the destruction of a large number of RBCs, it is essential to quickly supplement exogenous RBCs capable of effective oxygen transport while simultaneously halting the immune storm. Active treatment of the primary disease should be pursued concurrently with anemia correction. Management of primary disease contradictions should be approached judiciously, and minimally invasive techniques should be employed whenever possible to minimize collateral damage from surgery. Surgical treatment can be staged according to the need and urgency. Therefore, rapid diagnosis of immune hemolytic anemia is key to reducing the severity of its complications and mitigating the organ damage caused by these complications. This approach facilitates simultaneous treatment of the primary disease, improving overall patient outcomes.

This is a single case, and more cases are needed to validate the treatment. In this case, the authors have not performed a detailed imaging analysis for all organs and include this as one of the limitations. In the future, we will also investigate whether the autoimmune hemolysis reaction induced by drugs in different organ infections is specific. The clinical phenomena observed in this case report may inspire more in-depth immunological research to unravel how the immune system is abnormally activated in cases of biliary tract infections or stones. This could help in the development of new immunomodulatory drugs as well as in screening patients who are at high risk for an immune response, making it possible to intervene earlier.

## Author contributions

**Data curation:** Xian-Feng Wang.

**Investigation:** Jun Chen, Xian-Feng Wang, Jin Yu, Sheng Xu, Feng Zheng.

**Project administration:** Zhong-Kai Ni.

**Validation:** Jin Yu, Sheng Xu.

**Writing – original draft:** Jun Chen.

**Writing – review & editing:** Zhong-Kai Ni.
